# Severe food restriction activates the central renin angiotensin system

**DOI:** 10.14814/phy2.14338

**Published:** 2020-01-10

**Authors:** Aline Maria Arlindo De Souza, Andrea Linares, Robert C. Speth, Glenda V. Campos, Hong Ji, Deoclécio Chianca, Kathryn Sandberg, Rodrigo C. A. De Menezes

**Affiliations:** ^1^ Division of Nephrology & Hypertension Department of Medicine Georgetown University Washington DC USA; ^2^ Department of Pharmaceutical Sciences College of Pharmacy Nova Southeastern University Fort Lauderdale FL USA; ^3^ Departamento de Ciências Biológicas Instituto de Ciências Exatas e Biológicas Universidade Federal de Ouro Preto Ouro Preto Brazil

**Keywords:** angiotensin converting enzyme (ACE), angiotensin converting enzyme 2 (ACE2), caloric reduction, inadequate food intake, RAS‐Fingerprint^®^

## Abstract

We previously showed that 2 weeks of a severe food restricted (sFR) diet (40% of the caloric intake of the control (CT) diet) up‐regulated the circulating renin angiotensin (Ang) system (RAS) in female Fischer rats, most likely as a result of the fall in plasma volume. In this study, we investigated the role of the central RAS in the mean arterial pressure (MAP) and heart rate (HR) dysregulation associated with sFR. Although sFR reduced basal mean MAP and HR, the magnitude of the pressor response to intracerebroventricular (icv) microinjection of Ang‐[1‐8] was not affected; however, HR was 57 ± 13 bpm lower 26 min after Ang‐[1‐8] microinjection in the sFR rats and a similar response was observed after losartan was microinjected. The major catabolic pathway of Ang‐[1‐8] in the hypothalamus was via Ang‐[1‐7]; however, no differences were detected in the rate of Ang‐[1‐8] synthesis or degradation between CT and sFR animals. While sFR had no effect on the AT_1_R binding in the subfornical organ (SFO), the organum vasculosum laminae terminalis (OVLT) and median preoptic nucleus (MnPO) of the paraventricular anteroventral third ventricle, ligand binding increased 1.4‐fold in the paraventricular nucleus (PVN) of the hypothalamus. These findings suggest that sFR stimulates the central RAS by increasing AT_1_R expression in the PVN as a compensatory response to the reduction in basal MAP and HR. These findings have implications for people experiencing a period of sFR since an activated central RAS could increase their risk of disorders involving over activation of the RAS including renal and cardiovascular diseases.

## Graphical abstract

In this study, we investigated the role of the central RAS in the mean arterial pressure (MAP) and heart rate (HR) dysregulation associated with sFR. A major finding of this study was that central administration of the AT1R antagonist, losartan, reduced the blood pressure after 2 weeks on a sFR diet in Female rats and radioligand binding to the AT1R was selectively increased in the PVN of sFR rats.

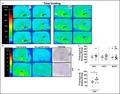

## 1 INTRODUCTION

Food restriction that does not lead to malnourishment is associated with longer life span in many species including humans (Anton & Leeuwenburgh, 2013). Intermittent fasting is another type of caloric restriction that has demonstrated health benefits including cardiac resilience to ischemia (Ahmet, Wan, Mattson, Lakatta, & Talan, 2005). Beyond a certain threshold, however, food restriction causes harm. Starvation damages multiple organ systems leading rapidly to death (Szafranski & Mekhail, 2014). Malnutrition from insufficient caloric consumption can also lead to death when the period of inadequate food intake is sustained.

Many people experience a cycle of inadequate food intake either voluntarily and involuntarily for different reasons. There are individuals who intentionally restrict their diet to reduce their body weight (BW) for their profession (e.g., models, gymnasts) or for psychiatric reasons (e.g., anorexia nervosa; DiVasta et al., 2010). Poverty is an involuntary cause of inadequate food intake. Based on annual surveys by the United States Department of Agriculture, nearly 16 million individuals living in the U.S. do not have enough to eat and lose weight as a consequence of very low food security (Coleman‐Jensen, Rabbitt, Gregory, & Singh, 2017). Natural disasters and wars have also led to periodic famine in large numbers of people (Muller & Krawinkel, 2005; Victora, Vaughan, Kirkwood, Martines, & Barcelos, 1986).

Inadequate food intake is associated with blood pressure dysregulation. A major clinical finding in individuals with anorexia nervosa is low blood pressure (BP) and reduced heart rate (HR; Sachs, Harnke, Mehler, & Krantz, 2016). However, the mechanisms underlying the association of inadequate food intake and blood pressure dysregulation are not fully understood.

The renin angiotensin system (RAS) is a key regulator of BP. In the periphery, the octapeptide angiotensin‐[1‐8] (Ang‐[1‐8]) modulates BP by binding to angiotensin type 1 receptors (AT_1_Rs) in the vasculature to cause vasoconstriction. We have been studying a rat model of severe food restriction (i.e., caloric intake is 40% of nonfood restricted animals; sFR). Animals lose approximately 15% of their normal BW over the 2 weeks period of sFR. Recently, we showed the RAS was activated in the periphery of female rats after 2 weeks on the sFR diet (de Souza et al., 2018). Plasma Ang‐[1‐8] was increased as well as the activity of angiotensin converting enzyme (ACE), which produces Ang‐[1‐8] from its inactive precursor Ang‐[1‐10]. AT_1_R mRNA expression was also increased in the microcirculation. These findings suggested that activation of the systemic RAS contributes to the BP dysregulation associated with inadequate caloric intake.

The RAS is also present in the brain (Premer, Lamondin, Mitzey, Speth, & Brownfield, 2013). Many studies show that Ang‐[1‐8] increases BP by directly acting on AT_1_Rs in the brain (Davisson, Oliverio, Coffman, & Sigmund, 2000; Nakamura, Yamazato, Ishida, & Ohya, 2017). Stimulation of brain AT_1_Rs modulates BP by causing thirst and subsequent water intake (Davisson et al., 2000) and via activation of the sympathetic nervous system (Blankestijn & Rupp, 2008; Pellegrino, Schiller, Haack, & Zucker, 2016). A central site of brain AT_1_R activity is the hypothalamus, which is a major regulator of fluid balance and BP regulation (Szczepanska‐Sadowska, Czarzasta, & Cudnoch‐Jedrzejewska, 2018). Thus, in this study, we determined the impact of sFR on the central RAS. We investigated BP and HR responses to ventricular microinfusion of Ang‐[1‐8] and to an antagonist of the AT_1_R. We also measured the effect of sFR on the enzymes and peptides involved in Ang‐[1‐8] metabolism and we assessed how sFR impacted AT_1_R binding in the hypothalamus by receptor autoradiography.

## 2 METHODS

### 2.1 Ethical approval

All experiments involving animals and maintenance were performed in accordance with the NIH Guide for the Care and Use of Laboratory Animals, European Convention for the Protection of Vertebrate Animals used for Experimental and other Scientific Purposes and according to the journal policies and regulations on animal experimentation. All efforts were made to follow the three Rs (Replacement, Reduction, and Refinement) and to avoid any unnecessary distress to the animals. All procedures were approved by the Animal Care and Use Committee (no. ‐ 2016/02; no. 16:1234).

### 2.2 Reagents

Ang‐[1‐8], Ang‐[1‐10], lisinopril, losartan, PD123319 were purchased from Sigma. Isoflurane was purchased from Cristália Ltda and Patterson Veterinary, Ketoprofen was purchased from Mundo Animal, ketamine (80 mg/kg) and xylazine (7 mg/kg). Penicillin, streptomycin, and dihydrostreptomycin were purchased from Fort Dodge Animal Health. Mca‐Ala‐Pro‐Lys(Dnp)‐OH was purchased from Enzo Life Science. MLN‐4760 was purchased from EMD Milllipore. Pierce BCA protein assay kit was purchased from ThermoFischer. ^125^I‐SI‐Ang‐[1‐8] was radiolabeled by Robert C. Speth, Ph.D. (Peptide Radioiodination Shared Resource, Georgetown University).

### 2.3 Animals

All experiments were conducted on female Fischer 344 rats (Envigo) initially weighing 180–190 g at 3 months of age. All animals were housed in individual cages on a 12 hr light–dark cycle at room temperature (24°C).

### 2.4 Diet

Two weeks after arriving at the animal facility, rats were single housed and food intake and BW were determined daily at 5:00 p.m. for 2 weeks. After this period, the animals were randomly divided into a control (CT) and severe food restriction (sFR) group. There were no differences in body weight (BW) between these two groups at the outset of the experiment [BW (g): CT, 201 ± 3 (*n* = 22) vs. sFR, 207 ± 2 (*n* = 24)]. All animals had ad libitum access to water and received a standard rat diet (Rodent diet 20, #5053, LabDiet) composed of 25% protein, 62% carbohydrate, and 13% fat. The CT group had free access to food for the duration of the study period. The daily amount of food given to the sFR group was 40% of the daily average of their previous 2‐week consumption. BW was measured daily before replenishing the food, as we previously described (de Souza et al., 2015).

### 2.5 Guide cannula surgery implantation

One week after beginning the dietary regimen, rats were first anesthetized with ketamine (80 mg/kg) and xylazine (7 mg/kg) i.p. and then placed in a stereotaxic frame (Stoelting Co.) with the incisor bar fixed 3.3 mm below the level of the interaural line. A unilateral guide cannula (23G, BDprecisionGlide^PM^, Becton Dickinson) was inserted into the lateral ventricle (LV) (10 mm) by stereotaxic surgery. The cannula was positioned using the Bregma as the reference point and by following the coordinates adapted from Paxinos & Watson Atlas (Paxinos & Watson, 2007) for the LV (−0.9 mm posterior; +1.1 mm lateral; −3.2 mm ventral). The guide cannula was secured by two screws and dental acrylic. After a dummy‐cannula (PlasticsOne) was inserted into the guide, the rats were returned to their home cages for recovery. After surgery, analgesics (4 mg/kg Ketoflex, 0.1 ml/300 g, s.c. and antibiotics (0.2 ml/100 g, penicillin, streptomycin, dihydrostreptomycin, s.c.) were administered.

### 2.6 Catheter implantation

Two weeks after beginning the dietary regimen, rats were anesthetized with isoflurane (2.5% at 3 L/min O_2_) and a polyethylene catheter was inserted into the femoral artery for cardiovascular measurements, as described previously (Loss Ide et al., 2007). The catheters were tunneled subcutaneously and exteriorized at the back of the neck. After surgery, analgesics (4 mg/kg Ketoflex, 0.1 ml/300 g, s.c.) and antibiotics (0.2 ml/100 g, penicillin, streptomycin, dihydrostreptomycin, s.c.) were administered. Experimental procedures began 48 hr after recovery from the anesthesia.

### 2.7 Microinjections

Two hours before microinjections, the rats were brought to the experimental room and remained in their home cages to minimize stress (Muller‐Ribeiro et al., 2012). All microinjections were performed during the light cycle in a room maintained at 24–25°C. Once HR and MAP were stable for at least 10 min, vehicle or drugs were microinjected into the lateral ventricle using a microinjector (30‐gauge, dental needle; PROCARE^®^ Lamedid Com LTDA) that was 1 mm longer than the guide cannula. The microinjector was connected to a 2 μl Hamilton syringe (Hamilton Robotics) filled with deionized water and joined to Teflon tubing (ID 0.12 mm; OD 0.65 mm; Bioanalytic Systems) filled with vehicle or drug. The microinjection was considered successful if the bubble present between the deionized water and drug was moving during the injection and if immediately after removal of the microinjector, flow appeared. The dose used for Ang‐[1‐8] (25 pmol) was a dose reported to cause a pressor response around 30 mmHg and stimulate the drinking behavior in conscious control rats when microinjected into the lateral ventricle. The dose for Losartan (24 nmol; Bunting & Widdop, 1995) was selected based in a dose reported to block the drinking behavior and pressor effect caused by icv Ang‐[1‐8] microinjection, usually 1,000 times higher than Ang‐[1‐8] (Beresford & Fitzsimons, 1992; Picard, Chretien, & Couture, 1995).

### 2.8 MAP and HR

MAP and HR were recorded continuously from the arterial catheter connected to a pressure transducer (MLT0699; ADI Instruments) and a signal amplifier (ETH‐400; CB Sciences Inc.). The analog signal from the amplifier was digitized by a 12‐bit analog‐to‐digital converter (PowerLab/400; ADI Instruments). The pulsatile arterial pressure was recorded at 1,000 Hz using Chart software (version 7.0 for windows, ADI Instruments). MAP and HR were derived online from the pulsatile arterial pressure measurements using pulse‐to‐pulse analysis (Gomide et al., 2013). These data were reported as the mean ± standard error of the mean (*SEM*) calculated from continual 2 min averages. Baseline values for MAP and HR were obtained by averaging the values of the 2 min‐period that preceded vehicle or drug injections.

### 2.9 Sample collection

To confirm injection site accuracy, rats were deeply anesthetized with ketamine (80 mg/kg) and xylazine (7 mg/kg) i.p. and subjected to transcardial perfusion with saline followed by 4% buffered paraformaldehyde in 0.1 M phosphate‐buffered saline (PBS). The brain was quickly removed, stored in 4% buffered paraformaldehyde overnight, then transferred to a 30% sucrose solution until saturated. Coronal sections (40 μm) were cut on a cryostat (Leica CM 1850, Leica Biosystems Inc.). Sections were mounted onto slides, air dried, and then counterstained with 1% neutral red solution and cover slipped. Sites of injections were approximated using the Atlas of Paxinos and Watson (George Paxinos, 2013).

For receptor autoradiography, rat brains were removed from anesthetized rats and placed in a rat brain matrix (Alto coronal brain matrix for small rats, Roboz Surgical Co) and kept at −20°C for 30 min before storing at −80°C until use. Cryostat (Leica CM 1850, Leica Biosystems Inc.) cut coronal sections were mounted onto positive charged slides in three replicates (for histology, nonspecific binding (NSB) and total binding).

For Ang‐[1‐8] metabolism and enzyme assays, after the rats were anesthetized with isoflurane (2.5% at 3 L/min O_2_), the hypothalamus was rapidly dissected and treated with a proprietary mixture of protease inhibitors (Attoquant^®^) and snap frozen in liquid nitrogen before storing at −80°C until use.

### 2.10 Ang‐[1‐8] metabolism

The hypothalamus was homogenized in phosphate‐buffered saline (PBS) using low‐energy sonication on ice. The concentration of protein in the homogenates were determined by the Pierce BCA protein assay kit (ThermoFischer). Ang‐[1‐8] metabolism was determined by liquid chromatography and tandem mass spectroscopy (LC‐MS/MS) as previously described (de Souza et al., 2018). In brief, tissue homogenates were spiked with Ang‐[1‐8] (100 ng/µg protein). After a 60 min incubation at 37°C, further metabolism was prevented by the addition of a proprietary peptidase inhibitor mix (Attoquant^®^). Angiotensin metabolites were then quantified by LC‐MS/MS using stable isotope‐labeled internal standards for each angiotensin metabolite at a concentration of 200 pg/ml. Following C18‐based solid‐phase‐extraction, samples were subjected to LC‐MS/MS analysis using a reverse‐phase analytical column (Acquity UPLC^®^ C18, Waters) operating in line with a XEVO TQ‐S triple quadrupole mass spectrometer (Waters) in multiple reaction monitoring mode. Internal standards were used to correct for peptide recovery of the sample preparation procedure for each angiotensin metabolite in each individual sample. Angiotensin peptide concentrations were calculated considering the corresponding response factors determined in appropriate calibration curves in original sample matrix, on condition that integrated signals exceeded a signal‐to‐noise ratio of 10. All angiotensin peptides were below the level of detection (<signal‐to‐noise ratio of 10) before spiking the samples with Ang‐[1‐8].

### 2.11 ACE activity

Ang‐[1‐10] (70 ng/µg protein) was added to the hypothalamic homogenates (10 µg protein) at 37°C in the presence or absence of the ACE inhibitor lisinopril (10 µM). Ang‐[1‐8] was measured as a function of time (over 60 min) by LC‐MS/MS. Total enzyme activity was measured in the absence of enzyme inhibitor. Non‐ACE activity was defined as peptidase activity measured in the presence of lisinopril. Specific ACE activity was defined as total enzyme activity minus non‐ACE activity and reported as pg/ml/hr (Kovarik et al., 2017).

### 2.12 ACE2 activity

ACE2 activity was measured after adapting a fluorogenic assay (Liu et al., 2010) for hypothalamic homogenates using the fluorogenic substrate Mca‐Ala‐Pro‐Lys(Dnp)‐OH. The protein concentration was determined by Pierce BCA protein assay kit (ThermoFischer). Reactions were conducted in 96 well microtiter plates containing 85 μL of Reaction Buffer in the presence of vehicle, the ACE inhibitor, captopril (20 µmol/L) and/or the ACE2 inhibitor MLN‐4760 (20 µmol/L). 10 μl of fluorogenic substrate was added to each well containing 40 μg of sample protein in 10 μl to achieve a final concentration of 30 μmol/L substrate. Product formation was immediately determined at 37°C by following the fluorescence as a function of time using a fluorescence plate reader (FLUOstar Omega, BMG LABTECH Inc.) at an excitation wavelength of 320 nm and an emission wavelength of 410 nm. Initial velocities were determined from the rate of fluorescence increase over the 100 min time course, which was in the linear range of the assay. Total enzyme activity was measured in the presence of vehicle (Reaction Buffer). Non‐ACE activity was defined as peptidase activity measured in the presence of captopril (20 µmol/L). Nonspecific peptidase activity was defined as the enzyme activity measured in the presence of captopril and MLN‐4760. Specific ACE2 activity was defined as non‐ACE activity minus nonspecific peptidase activity.

### 2.13 Receptor autoradiography

Slides containing hypothalamic and ventricle sections were incubated with ^125^I‐sarcosine^1^, isoleucine^8^‐Ang‐[1‐8] (^125^I‐SI Ang‐[1‐8]) to radiolabel angiotensin type 1 receptors (AT_1_Rs). Adjacent slides were separated into two sets: 'total binding' with no AT_1_R antagonist and 'nonspecific binding' (NSP) in the presence of a receptor saturating concentration of a nonradiolabeled AT_1_R antagonist (losartan, 10 µM). A saturating concentration of a nonradiolabeled angiotensin type 2 receptor antagonist (PD123319, 10 μM) was also present in the incubation buffer to prevent ^125^I‐SI‐Ang‐[1‐8] binding to the AT_2_R subtype. During a 30 min preincubation at ~22°C, NSP slides were exposed to 10 μM PD123319 and losartan, while 'total binding' slides were exposed to 10 μM PD123319. Slides were then incubated with ^125^I‐SI‐Ang‐[1‐8] in the presence of PD123319 for 'total binding', and PD123319 plus losartan for NSP in assay buffer, followed by several 'washes' in buffer, and water to remove salts and nonspecifically bound radioligand. The slides were dried, then exposed to autoradiography film using MR1 autoradiography film (Carestream) in an X‐Ray cassette. The film was developed and the images were scanned into a computer for quantitative densitometry using the MCID analysis software 7 (Imaging Research Inc.). The histology slides were thionin‐stained as described (Linares, Couling, Carrera, & Speth, 2016).

### 2.14 Statistical analysis

Prism software (version 7.0, GraphPad Software) was used to analyze all data and to construct the graphs. The data are expressed as mean ± *SEM*. The results obtained from receptor autoradiography were compared using a paired Student's *t* test to keep samples with the same radiation half‐life level. The data for basal parameter characterization, enzyme activity, and peptide concentrations were analyzed first for normality using the Shapiro‐Wilk normality test and then analyzed using the Student's unpaired *t* test to assess differences between groups. All MAP or HR responses to drug stimulation were compared by two‐way (time and diet as factors) analysis of variance (ANOVA) followed by Bonferroni post‐test using all the time‐points showed on the graph. All the results were tested for outliers considering (Mean*2 ± *SD*). The significance threshold level was set at 0.05.

## 3 RESULTS

### 3.1 Animal model

**Figure 1:**
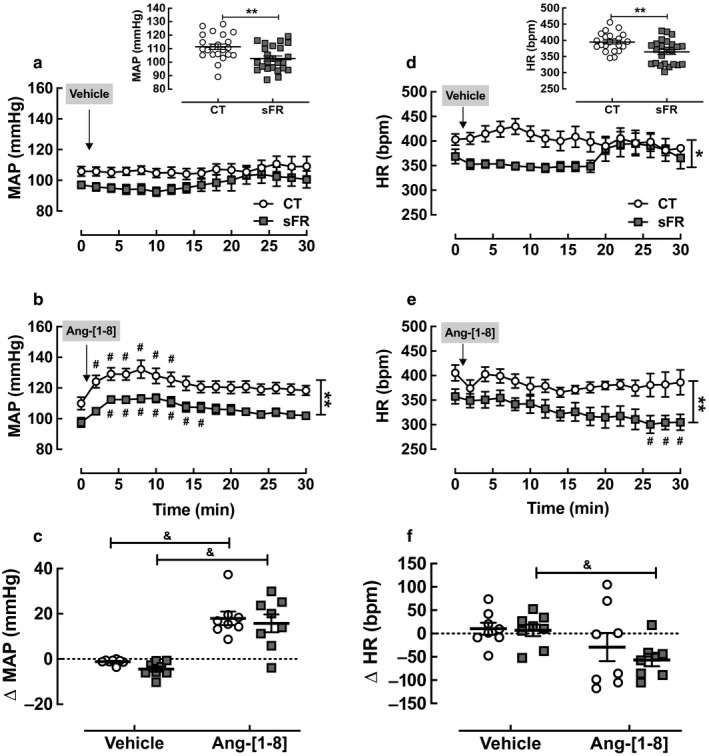
Effect of severe food restricted (sFR) on mean arterial pressure (MAP) and heart rate (HR) responses to icv injection of Ang‐[1‐8]. Shown is the basal MAP (a, *Inset*) and HR (d, *Inset*) from CT (*n* = 22) and sFR (*n* = 24) rats and MAP (a, b, c) and HR (d, e, f) after icv microinjection of vehicle and Ang‐[1‐8] in CT (open circle; *n* = 8) or sFR (closed square; *n* = 8) animals. The peak Ang‐[1‐8] response for MAP (c) and HR (f) were calculated at 10 and 26 min, respectively, after subtracting baseline (*t* = 0) measurements. *p < .05 and **p < .01 versus CT, same treatment either by Student's *t* test or two‐way ANOVA repeated measures indicated by vertical bracket (MAP Ang‐[1‐8]: Diet: *F* = 166, DFn = 1, DFd = 221; Time: *F* = 4.1, DFn = 15, DFd = 221; Interaction: *F* = 0.1, DFn = 15, DFd = 221); (HR Ang‐[1‐8]: Diet: *F* = 87, DFn = 1, DFd = 221; Time: *F* = 1.3, DFn = 15, DFd = 221; Interaction: *F* = 0.5, DFn = 15, DFd = 221); (HR vehicle: Diet: *F* = 48, DFn = 1, DFd = 177; Time: *F* = 0.5, DFn = 15, DFd = 177; Interaction: *F* = 1.4, DFn = 15, DFd = 177); ^#^ *p* < .05 versus baseline same group, same treatment by Student's *t* test; ^&^ *p* < .05 versus vehicle, same group by Student's *t* test. Values are expressed as the mean ± *SEM*

Before starting the 2 weeks sFR diet regimen, there were no differences between CT and sFR animal groups in initial BW [(g): CT, 201 ± 3 (*n* = 22) vs. sFR, 207 ± 2 (*n* = 24)]. Similarly to our previous findings (de Souza et al., 2018), 2 weeks of the sFR diet caused a 12% loss in final BW [(Final‐Initial (g)): CT, −0.1 ± 1.9 vs. sFR, −24.7 ± 1.7; *p* < .001]. Terminal measurements assessed by indwelling catheters showed the MAP was 9 mmHg lower (Figure 1a, *inset*) and HR was 30 bpm lower (Figure 1d, *inset*) in sFR rats when compared to animals fed a CT diet.

### 3.2 MAP and HR responses to central administration of Ang‐[1‐8]

To assess the effect of sFR on central Ang‐[1‐8] responses, rats instrumented with indwelling catheters were microinjected with 0.1 µl of Ang‐[1‐8] (25 pmol) into the lateral ventricle. We infused 25 pmol of Ang‐[1‐8] by icv microinjection because this dose administered icv was shown to cause a 30 mmHg rise in mean blood pressure and to stimulate drinking behavior in conscious rats (Picard et al., 1995). Vehicle had no effect on MAP in either the CT or sFR groups (Figure 1a). Ang‐[1‐8] increased the MAP in both the CT and sFR groups when compared to baseline (Figure 1b) or vehicle (Figure 1c). In both CT and sFR groups, peak MAP responses to Ang‐[1‐8] occurred by 10 min before gradually returning to baseline over the following 10 min. No differences were observed in the magnitude of the MAP response to Ang‐[1‐8] between the CT (18 ± 3 mm Hg) and sFR (16 ± 4 mm Hg) groups (Figure 1c).

There was no effect of vehicle on HR (Figure 1d). Microinjection of Ang‐[1‐8] had no detectable effect on HR in the CT group when compared to baseline (Figure 1e). In contrast, the peptide gradually lowered HR over 26 min in the sFR animals when compared to baseline or to the CT rats (Figure 1e) and resulted in a significant drop in HR (−57 ± 13 bpm; Figure 1f).

### 3.3 MAP and HR responses to central administration of losartan

**Figure 2:**
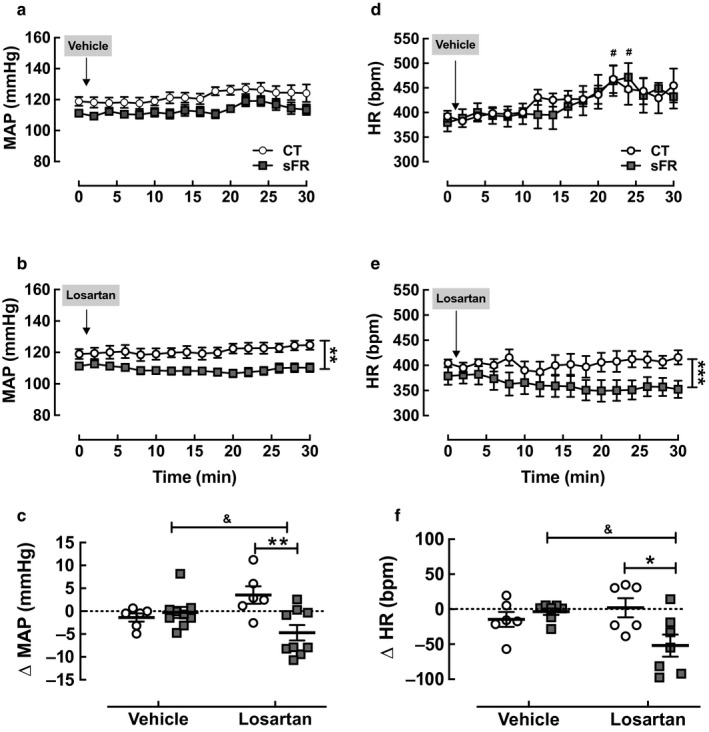
Effect of severe food restricted (sFR) on MAP and heart rate (HR) responses to icv injection of losartan. Shown is the MAP (a, b, c) and HR (d, e, f) after icv microinjection of vehicle and losartan in CT (open circle; *n* = 6) or sFR (closed square; *n* = 9) animals. The maximum losartan response for MAP (c) and HR (d) were calculated at 20 min after subtracting baseline (*t* = 0) measurements. *p < .05, **p < .01, and ***p < .001 versus CT, same treatment either by Student's *t* test or two‐way ANOVA indicated by vertical bracket (MAP Losartan: Diet: *F* = 129, DFn = 1, DFd = 206; Time: *F* = 0.4, DFn = 15, DFd = 206; Interaction: *F* = 0.4, DFn = 15, DFd = 206); (HR Losartan: Diet: *F* = 34, DFn = 1, DFd = 208; Time: *F* = 0.2, DFn = 15, DFd = 208; Interaction: *F* = 0.3, DFn = 15, DFd = 208); ^#^ *p* < .05 versus baseline same group, same treatment by Student's *t* test; ^&^ *p* < .05 versus vehicle, same group by Student's *t* test. Values are expressed as the mean ± *SEM*

To further assess the effect of sFR on the brain RAS, rats instrumented with indwelling arterial catheters were microinjected with 1 µl of the AT_1_R antagonist losartan (24 nmol) into the lateral ventricle. We infused 24 nmol of losartan because this dose was shown to block both the pressor and drinking behavior effects of Ang‐[1‐8] (Bunting & Widdop, 1995). There was no effect of vehicle (Figure 2a) or losartan (Figure 2b) on MAP in the CT group when compared to baseline or vehicle (Figure 2c). In contrast, losartan reduced MAP in the sFR group when compared to baseline (Figure 2b) or vehicle (Figure 2c). The AT_1_R antagonist gradually decreased MAP over 20 min in the sFR rats compared to CT rats leading to a maximum reduction of 5 mmHg (Figure 2b).

Microinjection of vehicle caused a small transient increase in HR in both the CT and sFR groups when compared to baseline (Figure 2d). Losartan had no effect on HR in the CT animals; however, it caused a gradual decrease in HR when compared to baseline (Figure 2e) leading to a maximum reduction in HR of 52 bpm at 20 min (Figure 2f).

### 3.4 ACE and ACE2 activity in the hypothalamus

**Figure 3:**
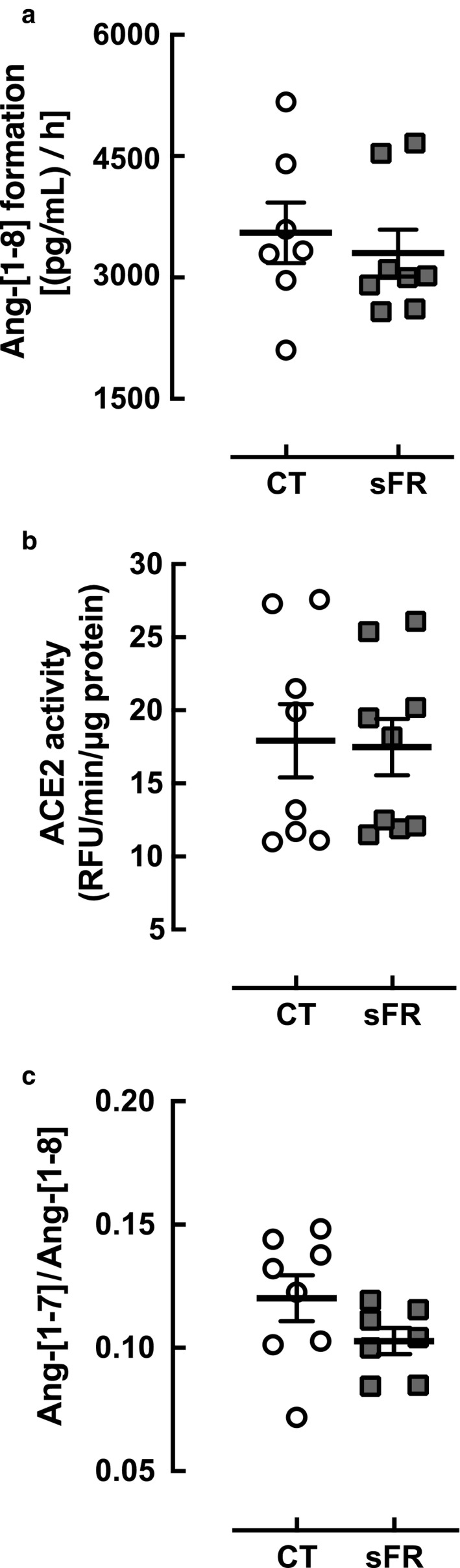
Effect of severe food restricted (sFR) on hypothalamic ACE and ACE2 activity. Shown are enzymes activity expressed as a rated of product formation for angiotensin converting enzyme (ACE) (a) (CT, *n* = 7; sFR, *n* = 8) and ACE2 (b, c) (CT, *n* = 8; sFR, *n* = 9) in hypothalamic homogenates from CT (open circle) or sFR (closed square) or the ratio of product to substrate (c). Values are expressed as the mean ± *SEM*

A rate limiting step in Ang‐[1‐8] production is the cleavage of two amino acids from the carboxy terminal of the decapeptide Ang‐[1‐10] (Paz Ocaranza et al., 2019). Thus, we investigated the effect of sFR on ACE activity in the hypothalamus by following the production of Ang‐[1‐8] over time by LC/MSMS (Figure 3a). No differences in the rate of Ang‐[1‐8] production by hypothalamic ACE were detected between CT and sFR animals.

Ang‐[1‐8] is catabolized by ACE2, which removes one amino acid from the carboxy terminus to form Ang‐[1‐7] (Forrester et al., 2018). Thus, we investigated the effect of sFR on ACE2 activity in the hypothalamus by measuring the catabolism of the fluorogenic substrate Mca‐Ala‐Pro‐Lys(Dnp)‐OH over time (Figure 3b) or by the Ang‐[1‐7]/Ang‐[1‐8] ratio after spiking samples with Ang‐[1‐8] (Figure 3c). No differences in hypothalamic ACE2 activity were detected between CT and sFR animals by either assay.

### 3.5 Levels of Ang‐[1‐8] and its metabolites in the hypothalamus

**Figure 4:**
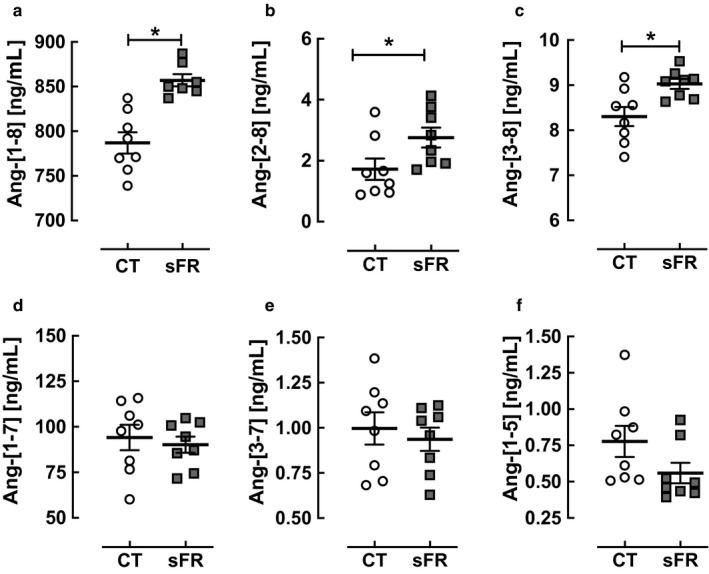
Effect of severe food restricted (sFR) on Ang‐[1‐8] metabolism in the hypothalamus. Shown are the levels of angiotensin (Ang) peptides after the addition of Ang‐[1‐8] to hypothalamus homogenates from rats on a CT (open circle) (a) and sFR (closed square) diet (*n* = 8/group). (a) Ang‐[1‐8], (b) Ang‐[2‐8], (c) Ang‐[3‐8], (d) Ang‐[1‐7], (e) Ang‐[3‐7] and (f) Ang‐[1‐5]. Values are expressed as the mean ± *SEM*

**Figure 5:**
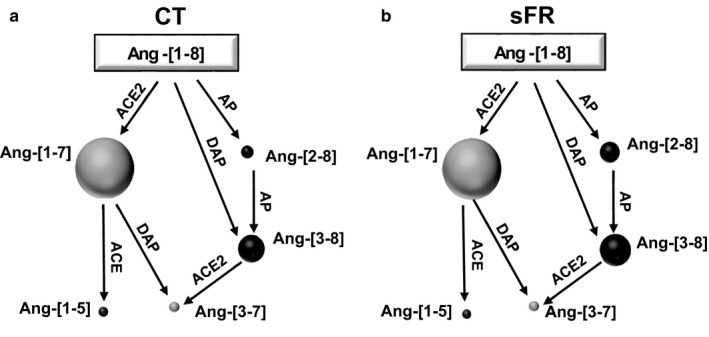
Representative figure of severe food restricted (sFR) on Ang‐[1‐8] metabolism in the brain stem and hypothalamus. Shown are representations of the relative amounts of Ang‐[1‐8] metabolites depicted by the circle volumes drawn to scale after the addition of Ang‐[1‐8] to hypothalamic homogenates from CT (a) and sFR (b) animals (*n* = 8/group). ACE, angiotensin converting enzyme; ACE2, angiotensin converting enzyme 2; AP, aminopeptidase; DAP, dipeptidyl aminopeptidase; NEP, neutral endopeptidase

To further assess the effects of sFR on Ang‐[1‐8] metabolism in the hypothalamus, Ang‐[1‐8] and its metabolites were measured by the RAS fingerprint^®^ method. The sFR diet increased the levels of Ang‐[1‐8] (1.1‐fold; *p* < .001; Figure 4a) and the metabolites Ang‐[2‐8] (1.6‐fold; *p* < .05; Figure 4b) and Ang‐[3‐8] (1.1‐fold; *p* < .01; Figure 4c). Other Ang‐[1‐8] metabolites including Ang‐[1‐7], were unaffected (Figure 4d‐f). The image representing the relative amounts of RAS peptides determined in the fingerprint assay shows the major catabolic pathway of Ang‐[1‐8] in the hypothalamus is through Ang‐[1‐7] in both CT (Figure 5a) and sFR (Figure 5b) rats.

### 3.6 AT_1_R binding in the brain

**Figure 6:**
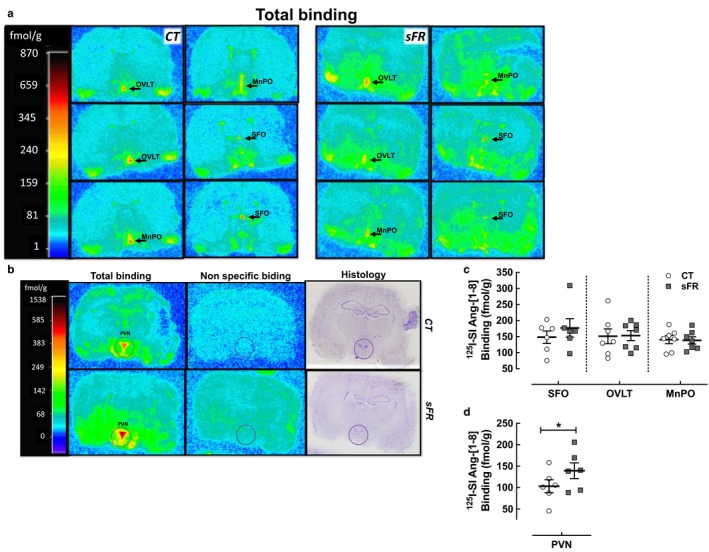
Effect of severe food restricted (sFR) on AT_1_R binding in the AV3V and hypothalamus. Shown are representative images of total and nonspecific ^125^I‐[Sar^1^,Ile^8^]‐angiotensin‐[1‐8] (^125^I‐SI‐Ang‐[1‐8]) binding by in vitro autoradiography to brain slices containing the periventricular anteroventral third ventricle (AV3V) (a) and hypothalamus (b) on a CT or sFR diet. (a) The first top left panels are approximately ~0.3 mm rostral to Bregma, while the last right bottom panels are ~0.4 mm caudal to Bregma. Midline regions expressing highest AT_1_R binding (red color) are the organum vasculosum of the lamina terminals (OVLT), median preoptic nucleus (MnPO), and the subfornical organ (SFO) moving from rostral to caudal sections. Other regions displaying high AT_1_ receptor binding include the piriform cortex, ventral medial preoptic nucleus, lateral preoptic nucleus, and the suprachiasmatic nucleus. (c) Quantitation of specific ^125^I‐[SI]‐Ang‐[1‐8] binding in CT (open circle) and sFR (close square) rats (*n* = 7/group) is shown in the periventricular anteroventral third ventricle (AV3V) (d) and in the PVN. *p < .05 versus CT, by Student's *t* test. Values are expressed as the mean ± *SEM*

To determine the effects of sFR on AT_1_R expression, we measured AT_1_R binding in the brain by receptor autoradiography in the periventricular anteroventral third ventricle (AV3V) and hypothalamus (Figure 6a,b). While there were no changes in AT_1_R binding in the SFO, OVLT, and MnPO of the AV3V (Figure 6c), 2 weeks of the sFR diet increased AT_1_R binding by 1.4‐fold in the PVN region of the hypothalamus (Figure 6d).

## 4 DISCUSSION

A major finding of this study was that central administration of the AT_1_R antagonist, losartan, reduced MAP after 2 weeks on a sFR diet but had no effect on MAP in the CT animals (Figure 2). These data indicate that sFR regulates MAP in part by activating the brain RAS. These findings extend our previous observation that administration of losartan into the bloodstream reduces MAP to a greater extent in sFR rats compared to the CT group (de Souza et al., 2018). The selective effect of central blockade of AT_1_Rs on MAP in sFR animals is similar to findings in the spontaneously hypertensive rat in a model of acute hemorrhage. Blocking central AT_1_Rs prior to inducing hemorrhage had a much greater depressor response in the spontaneously hypertensive rat compared to the WKY normotensive strain (Lee et al., 1995). These studies suggest that the sFR rat and the spontaneously hypertensive rat are both models of over activation of the brain RAS.

A second major finding was that radioligand binding to the AT_1_R was selectively increased in the PVN of sFR rats (Figure 6). The PVN has a high density of AT_1_Rs (Rowe, Grove, Saylor, & Speth, 1990; Song, Allen, Paxinos, & Mendelsohn, 1991; Tsutsumi & Saavedra, 1991) and is known to play a key role in regulating sympathetic activity (Chen & Toney, 2010). Microinjection of Ang‐[1‐8] into the PVN from conscious Wistar male rats was shown to increase BP by 13 mmHg and to also increase lumbar sympathetic nerve activity (Braga et al., 2011; Buttler, Ribeiro, Ferreira‐Neto, & Antunes, 2016). Furthermore, compared to Wistar‐Kyoto (WKY) rats, the PVN of spontaneously hypertensive rats have higher AT_1_R mRNA expression (Agarwal, Welsch, Keller, & Francis, 2011) and greater ^125^Sarcosine^1^ Ang‐[1‐8] binding (Gutkind, Kurihara, Castren, & Saavedra, 1988). Increased AT_1_R binding in the PVN would increase sympathetic outflow and raise MAP (Dampney, 1994). Thus, the up‐regulation of AT_1_Rs in the PVN is a likely compensatory response to the depressor effects of hypovolemia‐induced by sFR (de Souza et al., 2018). This higher AT_1_R expression also could contribute to the previously observed increase in adrenergic response in the vasculature (de Souza et al., 2015) since it is known that AT_1_Rs located in the PVN can stimulate the sympathetic response (Dampney, 1994). A study of male Sprague‐Dawley rats deprived of water for 48 hr showed evidence of AT_1_R activation in the PVN (Freeman & Brooks, 2007). Blood pressure gradually decreased after the PVN was microinjected with the AT_1_R antagonist candesartan. In contrast, candesartan had no effect on BP in the water‐replete control rats. Thus, increased sympathetic flow as a result of up‐regulation of AT_1_R activity in the PVN contributes to MAP maintenance in the sFR hypovolemic state.

We did not observe differences in ACE or ACE2 activity in hypothalamic homogenates between CT and sFR rats, suggesting that 2 weeks of sFR does not impact hypothalamic Ang‐[1‐8] metabolism through these metabolic enzymes. This finding is in contrast with our observations in the circulation where we found 2 weeks of sFR increased both ACE and ACE2 activity (de Souza et al., 2018). Thus, sFR may not regulate these metabolic enzymes centrally. Some studies have shown the brain RAS is regulated by other metabolic enzymes. Aminopeptidases such as aminopeptidase A, metabolize Ang‐[1‐8] to Ang‐[2‐8], Ang‐[3‐8], and Ang‐[4‐8] in the brain (Karamyan & Speth, 2007). Thus, a reduction in these other peptidases could explain the increased levels of Ang‐[1‐8] in the hypothalamus. However, the increase in Ang‐[2‐10] in the sFR group compared with CT rats could also suggest there is more aminopeptidase A in the sFR hypothalamus. Thus, we cannot rule out that sFR regulates the hypothalamic RAS through these other proteases.

The AT_1_R is known to be constitutively active (Unal & Karnik, 2014). Thus, the mere increase in AT_1_R expression in the PVN could be sufficient to generate a sufficient constitutive activity‐based signal to activate efferent pathways of the PVN leading to increased sympathetic nervous system activity independently of, or in addition to, agonist activation.

We previously showed that 2 weeks on a sFR diet increased plasma levels of Ang‐[1‐8] by 1.3‐fold (de Souza et al., 2018). Ang‐[1‐8] levels in the hypothalamus were below the detection limit of the RAS Fingerprint^®^ assay, which is one of the most sensitive methods available for detecting tissue levels of angiotensin peptides. However, there were higher levels of Ang‐[1‐8] after spiking hypothalamic homogenates with Ang‐[1‐8], which suggests that sFR leads to less catabolism of Ang‐[1‐8] in this brain region as noted in the previous paragraph. It is possible that Ang‐[1‐8] levels are much higher in the PVN in contrast with the rest of the hypothalamus and that the effect of sFR on Ang‐[1‐8] metabolism is diluted by the surrounding tissue. Unfortunately, the PVN is a too small brain region to measure angiotensin peptide levels with the technology presently available to us.

Interestingly, the injection of losartan icv led to a bradycardic response. The finding that administration of losartan icv led to a bradycardic response in sFR but not CT rats suggests that central AT_1_Rs play a greater role in HR regulation by loss of enhanced sympathetic activation. This is an important observation that needs to be more investigated. Similar response was observed in spontaneously hypertensive rats (SHR) which have greater sympathetic activity than normotensive WKY rats (Liang, Mitchell, Smith, & Mizuno, 2016) and losartan administered icv was shown to impair the tachycardic reflex response to hemorrhage only in SHR compared to WKY rats (Lee et al., 1995). Furthermore, there was a sympatho‐inhibitory influence of Ang‐[1‐8] mainly mediated by parasympathetic inputs, since the sympatho‐inhibitory actions of Ang‐[1‐8] are of greatest influence during hypotension (Head, Saigusa, & Mayorov, 2002).

Studies of food restriction vary tremendously from Starvation and malnutrition reduces lifespan (Szafranski & Mekhail, 2014) whereas food intake without malnutrition (mild food restriction) increases life expectancy (Fontana & Partridge, 2015; Testa, Biasi, Poli, & Chiarpotto, 2014). In this diet protocol, maintenance on the sFR diet would lead to mortality in 18 days (Avraham, Bonne, & Berry, 1996). What is not well‐studied is how exposure to a period of sFR affects health long term. Thus, it will be interesting in future studies to determine if the brain RAS returns to normal months after the sFR period has ended.

In conclusion, the increase in AT_1_R binding in the PVN and the reduction in sympathetic nervous system activity after central microinjection of losartan suggests sFR activates the brain RAS to compensate for the depressor response arising from the reduced plasma volume. Further studies are warranted to fully understand the brain pathways and other nuclei involved in these responses. These findings have implications for individuals who have experienced a period of sFR either voluntarily or involuntarily. Activation of the central RAS may increase their susceptibility to disorders of the RAS including renal and cardiovascular disease.

## CONFLICT OF INTEREST

None.

## AUTHOR CONTRIBUTIONS

Conception and design of the experiments: A.M.A.S., R.C.A.M., K.S.; collection, analysis and interpretation of data: A.M.A.S., G.C., A.L., R.C.S., H.J., D.C.J, K.S., R.C.A.M; drafting and revising the article critically for important intellectual content: A.M.A.S., G.C., A.L., R.C.S., H.J., D.C.J, K.S., R.C.A.M. All authors approved the final version of the manuscript. All the persons cited qualify for authorship and all the authors approved the final version of this manuscript.
